# Persistence of *Coffea arabica* and its relationship with the structure, species diversity and composition of a secondary forest in Brazil

**DOI:** 10.1371/journal.pone.0194032

**Published:** 2018-03-14

**Authors:** Diego Raymundo, Jamir Prado-Junior, Norberto Emídio de Oliveira-Neto, Lucas Dezidério Santana, Vagner Santiago do Vale, Tamiel Baiocchi Jacobson, Paulo Eugênio Alves Macedo de Oliveira, Fabrício Alvim Carvalho

**Affiliations:** 1 Institute of Biology, Federal University of Uberlândia, Uberlândia, Brazil; 2 Institute of Biological Sciences, Federal University of Juiz de Fora, Juiz de Fora, Brazil; 3 Department of Forest Sciences, Federal University of Lavras, Lavras, Brazil; 4 Forest Science Department, State University of Goiás, Campus Ipameri, Ipameri, Brazil; 5 Ecology Department, Brasília University, Brasília, Brazil; Technical University in Zvolen, SLOVAKIA

## Abstract

Understanding the relationships between *Coffea arabica* L. and the native tree community of secondary forests regrowing after the abandonment of coffee plantations is important because, as a non-native species in the Neotropics, coffee can outcompete native species, reducing diversity and forests ecosystem services. We aimed to answer three questions: 1) Does coffee regeneration in secondary forests differ between shaded and unshaded abandoned plantations?; 2) How is coffee basal area related to structural attributes, species diversity and composition of the native community?; and 3) Do the relationships between coffee and native community differ between tree and sapling components? We sampled the tree and sapling components in a seasonal tropical dry forest that were previously used as shaded and unshaded coffee plantations. Coffee was the most important species in the sapling component of shaded systems, but was almost absent in unshaded ones. Coffee basal area was negatively related with the native density and absolute species richness of the sapling component; and was negatively related with tree density, and positively related with the percentage of pioneer individuals of the native tree component. Our results indicate that coffee persists in secondary forest communities even after more than 70 years of shaded-coffee plantations were abandoned, potentially reducing density and diversity of native species. Despite limitations, which hinder more general conclusions on coffee invasiveness in Brazilian secondary tropical forests, our results indicate that coffee is a strong competitor in the studied secondary forests and provide important insights for future research on this topic.

## Introduction

Secondary forests account for most forest cover in the world [1], and their extension and importance are likely to increase in the future [2]. During secondary forest succession, a mix of native and non-native tree species often coexist and compete for the resources [3]. A shift in resource availability during succession can cause a shift in community composition and structure from secondary to old-growth forest [4]. Non-native species can play an important role during succession [5], as they often have strategies of rapid resource uptake and use and can, in some cases, outperform native species [6, 7]. A challenge is to understand the role of non-native species in the community processes because they can potentially become invasive [8–10]. Evaluating the relationships between native and non-native flora will help us to understand how anthropogenic activities can affect ecological processes in secondary forests, and how to manage these forests for conservation and sustainable agroforestry systems [11].

The association of secondary forests and *Coffea arabica* L. is widespread in tropical countries [12, 13]. In African forests, coffee is a long-lived native shrub species, adapted to the shaded understory condition and occurring in both secondary and old-growth forests [14]. In the Neotropics, coffee is a non-native species, mainly cultivated under two systems: 1) the traditional monoculture, where coffee is cultivated under full sun (i.e unshaded coffee plantations, without direct association with other tree species), or 2) under a canopy of native trees species to shade coffee plants (shaded coffee plantations), similar to the natural habitat of coffee in Africa [15–17]. Many of these plantations have been abandoned in recent decades, allowing the regeneration of secondary forests in which coffee plants persist [18]. Although the shaded environment tends to be achieved over secondary forests succession, coffee trees from shaded and unshaded plantations experience strong differences in environmental conditions during the first decades of regeneration, which can influence coffee population performance and persistence in those secondary forests. Because coffee trees from shaded plantations are under similar conditions to its natural habitat before plantation abandonment, it is expected that coffee persistence would be higher in secondary forests growing after abandonment of shaded coffee plantations.

A central but yet understudied question is whether coffee can affect Neotropical secondary forest communities. Coffee agroforestry systems provide important ecosystem services such as carbon storage [19], and habitat to flora and fauna species [20–22]. Conversely, studies in secondary forests showed an invasive potential of coffee, related to its capacity to establish and increase the populations in forest understories, leading to lower native species richness [23–25]. However, most studies have focused on comparisons between the attributes of old-growth forests (e.g. structure and floristic) and forests associated with coffee, without analysing the direct relationships between coffee and the multiple forests attributes [21, 26]. Moreover, they have focused on native tree component, neglecting the influence of coffee on the sapling component [27, 28]. As an understory species (rarely higher than 8 m), the influence of coffee should be stronger on saplings and seedlings than on mature trees. Thus, evaluating the sapling component of secondary forest growing after coffee plantation abandonment provides important information on their dynamics [25, 29, 30].

In addition to changes in forest structure and species richness, it is expected that coffee would affect forest composition (e.g. species ecological groups) [31]. For instance, changes in species composition can shift the ratio of pioneer/shade-tolerant species, which can directly affect forest dynamics, light requirements for successful regeneration, and forest aboveground biomass and carbon residence [32, 33]. The competitive effect of coffee on pioneer species should be stronger because coffee can shade the understory and limit the establishment of species that have high light requirements [34, 35]. As a consequence, a high population of coffee trees is likely to decrease the percentage of pioneer species in the community.

The aim of this study was to evaluate the relationship between *Coffea arabica* populations and the native communities (tree and sapling components) of secondary forests regrowing after the abandonment (> 70 years) of shaded and unshaded coffee plantations. We addressed three main questions: 1) Does coffee persistence in secondary forests differ between shaded and unshaded abandoned plantations? We hypothesized that after abandonment, the relative importance of coffee (i.e. density, basal area and frequency) would be higher in forests growing in previously shaded areas because their environmental conditions would be more similar to forests where coffee is native since the plantation abandonment, while unshaded coffee will experience the hash environmental of initial stages of forest succession before get fully shaded; 2) How is coffee basal area related to the structure, species diversity and composition of the native community? We hypothesized that coffee (a non-native species) would be negatively related with native forest attributes (*e*.*g*. species density, basal area, species richness, and percentage of pioneer individuals); 3) Do the relationships between coffee plants and the native community differ between tree and sapling components? We hypothesized that the relationship of coffee (an understory species) and the native community is size-dependent, being stronger for the sapling component than the tree component.

## Methods

### Study areas and species sampling

This study was conducted in three patches of seasonal dry forest in Juiz de Fora municipality, Minas Gerais state, Brazil. Two of them are located in the Botanical Garden of the Federal University of Juiz de Fora (BGJF-1 and BGJF-2, 21°43.894' S and 43° 22.354' W, 80 ha) and the other in the Mariano Procópio Park (MPP, 21° 44.732' S and 43° 21.547' W, 5 ha). Sampling permission in BGJF was granted by Federal University of Juiz de Fora and permission in MPP was granted by the administration of MPP. The distance between BGJF and MPP is 2.3 km and both areas are conservation units surrounded by an urban matrix. During the sampling, no signs of cattle grazing, fire and/or selective logging were observed. These forests experience a homogeneous climate, defined as warm temperate climate (Cwb Megathermic climate of Köppen), with warm and rainy summers and dry winters. Mean annual rainfall is 1516 mm and mean annual temperature is 19°C [36]. Altitude ranges from 700–760 m a.s.l., and soil type is predominantly Dystrophic Red-Yellow Latosol.

In the past (> 70 years ago), coffee cultivation was an important economic activity in the region of Juiz de Fora [37] and the study areas were covered by coffee plantations. Two of them (BGJF-1 and MPP) used the shaded coffee plantation system (native trees shading the coffee), and one (BGJF-2) used the unshaded coffee plantation system (traditional monoculture, where coffee is cultivated under full sun). In shaded areas, coffee was cultivated under a canopy of the native species *Piptadenia gonoacantha* (Fabaceae) due to the ability of this species to fix nitrogen, its longevity, and provide a broad canopy. The preparation of a shaded coffee plantation included the elimination of all the native vegetation with exception of relatively few canopy individuals. In the 1930s, Brazil experienced an economic crisis in coffee cultivation, and most of coffee plantations located in the region of Juiz de Fora were abandoned [37], allowing the regeneration of secondary forest patches that we used in this study. Unfortunately, satellite or aerial images that could help us to show the original coffee plantations are not available, considering that they were abandoned > 70 years ago, and the only sources of information on the age of the forest patches and past land-use history were interviews with local residents, landowners, based on official documents of BGJF and MPP, and previous studies in the area [37–39]. Although some farmers used to harvest old coffee trees for firewood prior to plantation abandonment, this was not the case for our study areas.

To evaluate the tree component, we allocated 25 randomized sample plots (20 x 20 m) in BGJF-1 (shaded coffee) and BGJF-2 (unshaded coffee), totalling 1-ha sample in each area. In MPP (shaded coffee) we allocated 10 randomized sample plots (20 x 20 m) totalling 0.4 ha sample. We sampled 10 plots instead of 25 plots in MPP because it is a small forest fragment (5 ha).We randomized sample plots in the core areas of each forest, so as to avoid edge effect in our plots. In each plot, the tree component included all living trees with stem diameter at breast height (DBH, 1.30 m) ≥ 5 cm. Trees were tagged, identified to species level and their diameter was measured. To evaluate the sapling component, sub-plots of 5 x 5 m were allocated inside each tree component plot (southeast corner), and all saplings with DBH ≤ 5 cm and height ≥ 1 m were tagged, identified to species level and their diameter was measured.

### Species ecological groups

Evaluating species into ecological groups is useful to understand how communities differ in species composition (e.g. successional stage) [33]. In forestry studies, species are usually classified according to their light requirements to establish and survive [32, 40]. Thus, we classified species into two successional groups according to the forest inventory data of Minas Gerais state [41] as pioneer species (P), that have high light requirements for successful establishment, growth and survival; and non-pioneer species (NP) that can establish and survive in shaded conditions. We acknowledge that non-pioneer (or shade tolerant species) can have a range of life strategies, as some have the ability to establish and survive in the shade during the whole life, whereas others establish in the shade but require a gap to grow to larger sizes [32]. However, most forestry studies have focused on the classical trade-off between pioneer species (fast-growing species with short life span) and shade tolerant species (slow-growing species with long life span) to explain ecosystem processes [42, 43]. Successional groups were available for most species sampled, covering 96% of sampled individuals. Additional information on species sampled and their classification into successional groups can be found in S1 Table.

### Data analysis

We evaluated the parameters of species relative importance values (RIV, i.e. relative density, basal area and frequency) in each forest patch and each community component. To test whether coffee regeneration RIV parameters differ between coffee cultivation systems (shaded vs unshaded) we used generalized linear mixed models (GLMM), including patch size as a random effect (to account for the possible confounding effect of patch size in our results). To account for the nestedness of plots within forest patches, we performed simultaneous autoregressive (SAR) models, assuming spatial autocorrelation among plots and including a second error term in the GLMM. SAR models evaluate the spatial dependence in the residuals of GLMM (SAR_error_), reducing or even removing the spatial correlation among plots [44, 45]. In SAR models, it is necessary to define a minimum weighted neighborhood structure to fit the spatial structure of the models residuals. We used a neighborhood distance of 0.003 decimal degrees (~300 m), because this was the maximum distance between plots within each forest patch, and sufficient to consider plots of different patches as non-neighbour plots [46]. To evaluate if SAR models removed the spatial autocorrelation, we performed Moran’s I tests for GLMM and SAR models. A p-value from Moran’s I test higher than 0.05 indicates that the model residuals do not show spatial autocorrelation.

To evaluate the relationships between coffee basal area and native forest attributes we used six parameters per plot including only native species: tree density (number of individuals), basal area (cm^2^), absolute species richness (S), rarefied species richness (S’), species diversity (Shannon-Wiener index, H’) and percentage of pioneer individuals (number of pioneers divided by total number of individuals). We used rarefied species richness to account for the confounding effect of tree density in species richness [47]. For rarefied richness we used 9 individuals, as this number was found in most plots in both tree and regeneration components (S2 Table). We calculated these parameters for the tree component and sapling component separately. To test how coffee is related with native flora, we performed bivariate relationship analysis between coffee basal area and native forest attributes, using GLMMs (including patch size as random effect) and SAR_error_ models. We used coffee basal area as a predictor because basal area better reflects species relative biomass, which is a better indicator of plant performance than abundance (i.e. the effect of one larger coffee tree is stronger than many coffee saplings). We did not include unshaded patch in the bivariate relationships between coffee and native forest attributes because coffee was absent in most plots (BGJF-2, see results section).

When necessary, data were square root transformed prior to analysis to meet the assumptions of normality, homoscedasticity, to reduce the effect of outliers, and to account for possible nonlinear relationships between variables. All analyses were performed using the platform R (R-Core-Team, 2015) and with the following packages: lme4 [48], vegan [49], spdep [50], and ggplot2 [51]. Additional information on plot structural, diversity, and composition attributes can be found in S2 Table.

## Results

**Fig 1:**
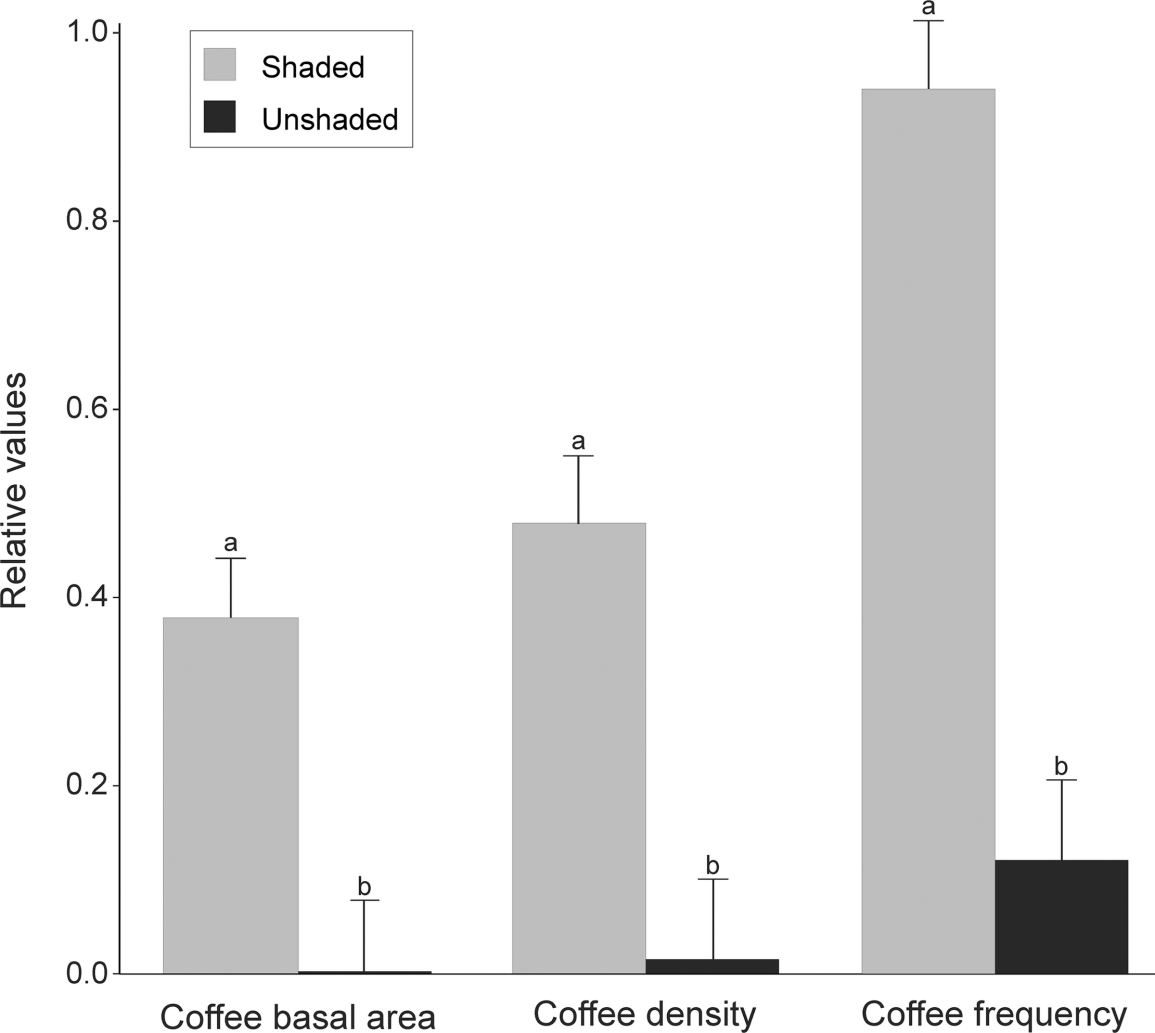
Comparisons of the relative basal area, density and frequency of coffee between shaded and unshaded systems that were abandoned (>70 years) and now are secondary forests in Juiz de Fora, Minas Gerais, Brazil. Area size was included as random effect to account for the possible confounding effect of patch size in our results and SAR models were performed to reduce spatial autocorrelation between plots. Different letters indicate significant differences between shaded and unshaded systems (p<0.05).

**Fig 2:**
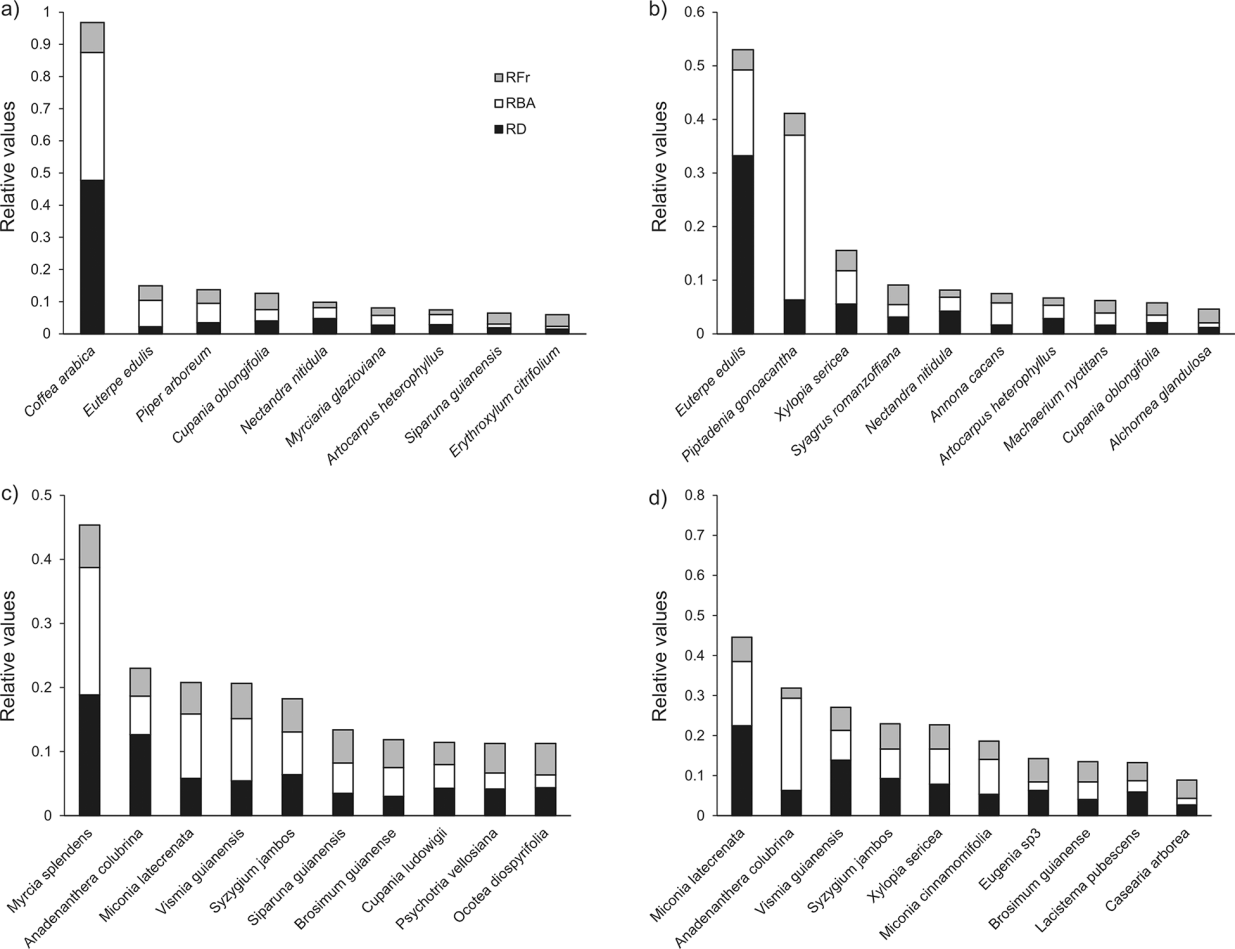
Relative basal area (RBA), relative tree density (RD) and relative frequency (RFr) of the 10 most important species for the a) sapling component of shaded systems; b) tree component of shaded systems; c) sapling component of unshaded system; and d) tree component of unshaded systems. Simultaneous autoregressive (SAR) models removed the spatial autocorrelation among plots in each regression analysis (Moran’I p-value > 0.05, Table 1). Bivariate relationships between coffee basal area and native community attributes differed between tree and sapling components. In the sapling component, coffee basal area was significantly negatively related with native saplings density (SAR regression coefficient β = -0.20) and absolute species richness (β = -0.56) (Fig 3C and 3E). In the tree component, coffee basal area was significantly negatively related with native tree density (β = -0.03) and positively related with the percentage of pioneer individuals (β = 0.002) (Fig 3D and 3L).

A total of 4,250 individuals (189 species and 48 families) were sampled in the tree component and 2,400 individuals (148 species and 40 families) in the sapling component (S1 Table). We found significantly lower values of coffee relative basal area, density and frequency in plots of the unshaded coffee system compared to plots of the shaded coffee system (Figs 1 and 2). Coffee was the most representative species in the sapling component of the shaded system, with 45% of individuals, 36% of basal area, and occurring in 93% of sample plots (Figs 1 and 2). Conversely, coffee had a small population in the sapling component of unshaded coffee system, with 2% of individuals, 0.4% of basal area, and occurring in 12% of the plots (Fig 1). In the tree component, only 32 individuals (< 0.01% of sampled individuals) of coffee were sampled in the three areas.

**Fig 3:**
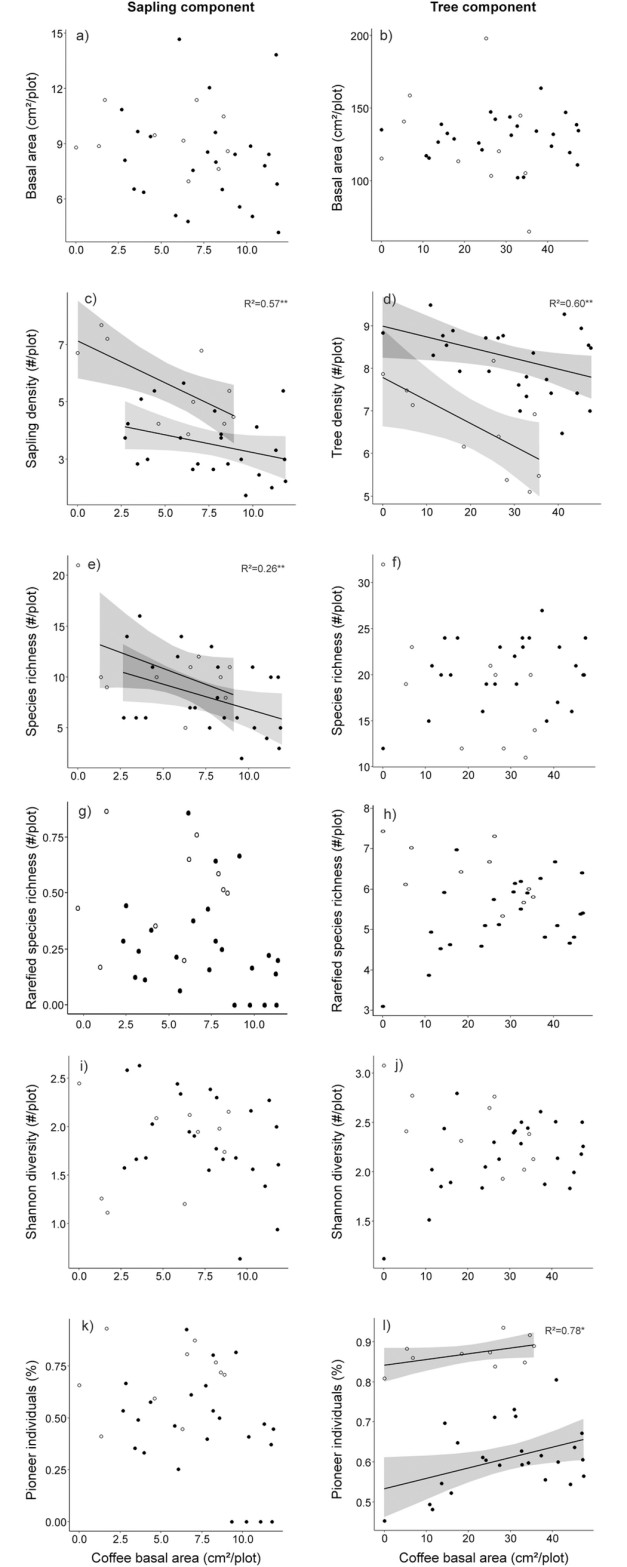
Bivariate relationships between coffee basal area and native community parameters of sapling and tree components. a) sapling basal area; b) tree basal area; c) sapling density; d) tree density; e) absolute species richness of saplings; f) absolute species richness of trees; g) rarefied species richness of saplings; h) rarefied species richness of trees; i) Shannon diversity of saplings; j) Shannon diversity of trees; k) percentage of pioneer saplings; l) percentage of pioneer trees. Only shaded systems were included because coffee was absent in most unshaded system plots (22 of 25 plots). Regression lines, confidence interval (95%) and Nagelkerke R^2^ of SAR models are given for both shaded sites (BGJF-1 –solid symbols, N = 25; and MPP–open symbols, N = 10). Sites were included as random effect to account for the nestedness of the plots within sites, and SAR models were performed to reduce spatial autocorrelation among plots.

**Table 1:** Simultaneous autoregressive (SAR) models between coffee basal area and native density (individuals.plot^-1^), basal area (cm^2^.plot^-1^), absolute species richness (species.plot^-1^), rarefied species richness (species.ind^-1^), Shannon diversity (H’.plot^-1^) of tree and sapling components. Moran’s I p-values > 0.05 indicate no spatial correlation among plots. The SAR models intercept, β coefficient, p-value and Nagelkerke R squared are given.

Parameter	Component	Intercept	β	p-value	Moran's I (p-value)	R^2^
Density	Tree	7.2	-0.029	**0.003**	0.663	**0.60**
	Sapling	6.6	-0.199	**0.001**	0.673	**0.57**
Basal area	Tree	129.7	-0.143	0.622	0.746	0.15
	Sapling	9.8	-0.096	0.492	0.632	0.13
Absolute species richness	Tree	18.9	-0.024	0.693	0.730	0.17
	Sapling	13.7	-0.569	**0.005**	0.455	**0.27**
Rarefied species richness	Tree	6.17	0.009	0.374	0.628	0.34
	Sapling	2.4	0.077	0.401	0.650	0.16
Shannon diversity	Tree	2.4	0.003	0.497	0.667	0.23
	Sapling	2	-0.038	0.161	0.466	0.08
Percentage of pioneers	Tree	0.8	0.002	**0.016**	0.384	**0.79**
	Sapling	0.7	-0.009	0.469	0.769	0.45

## Discussion

We evaluated how coffee populations are related to structural and diversity attributes of secondary forests growing after abandonment of shaded and unshaded coffee plantations. We found that coffee population persisted (after more than 70 years of abandonment) with high basal area, density, and frequency in the understory of shaded plantations but was almost absent in the unshaded plantation. We found that coffee was negatively related with native density (tree and sapling components), and absolute species richness (sapling component), and was positively related with the percentage of pioneer individuals (tree component).

### Previous coffee cultivation system (shaded and unshaded) influences the persistence of coffee population

We hypothesized that the relative importance of coffee would be higher in secondary forest growing in abandoned shaded plantations because coffee trees are under similar conditions to its native habitat since plantation abandonment. Conversely, although a shaded environment will be achieved in unshaded plantations areas after decades of abandonment, coffee trees experience a harsh environment during initial stages of forest succession before they get fully shaded. We indeed found higher coffee relative basal area, density and frequency in shaded plantations than unshaded plantations (Figs 1 and 2). Similar results were found in other studies evaluating secondary forests regeneration after coffee plantation abandonment, where coffee shows higher dominance in shaded systems than in unshaded systems or in open areas, such as pastures within a coffee plantation matrix [52–54]. When an unshaded coffee plantation is abandoned, many coffee trees may be outcompeted by native climbers, shrubs and early pioneers that aggressively colonize abandoned areas under full sunlight. Moreover, under full sun condition, coffee has a shorter life span and is highly dependent of external resource inputs, usually alternating between high and low production over the years [55, 56]. Therefore, coffee populations from unshaded plantations can be strongly reduced before they achieve the shaded environment of secondary forests in latter successional stages. On the other hand, when a shaded coffee plantation is abandoned, coffee trees are already under an established canopy and compete in a thinned and shaded understory, which favors coffee growth [53]. Moreover, under shaded conditions coffee usually has longer periods of fruit production and a more constant production every year (e.g. less sensitive to yearly rainfall variations than unshaded coffee). Shading trees can also enhance coffee performance in the shaded system by reducing local environmental stresses that constraint coffee growth (*e*.*g*. wind intensity, desiccation, temperature variation and soil degradation), and by increasing nutrient recycling through litter decomposition [57–59]. Finally, a higher number of species surrounding coffee plants can reduce (indirectly, through lower likelihood of growing near a conspecific individuals) the effect of host-specific pathogens and herbivores [60, 61], attract local fauna, and consequently coffee seed dispersers, thereby increasing coffee performance in shaded systems.

We acknowledge that differences in coffee performance under shaded and unshaded conditions could be related to differences in coffee varieties or cultivars, but unfortunately we do not have this accurate information. Yet, considering the interviews with local residents and landowners, the long-time interval since plantations were abandoned (>70 years), and that research on high-yielding and disease-resistant coffee varieties only occurred from the late 1960s, we assumed that coffee plants in all studied forest patches belong to the same cultivar of *Coffea arabica*.

Our results indicate that coffee is a strong competitor in secondary tropical forests growing after the abandonment of shaded plantations, and highlight the importance of understanding abandoned plantation legacies, because the species cultivated in the original system can persist for a long time and become “ghosts of cultivation past” [10].

### The negative relationships between coffee basal area and the native forest attributes

We hypothesized that coffee basal area (a non-native species in the Neotropics) would be negatively related with native forest attributes, and that these relationships would be size-dependent, being stronger for the sapling component (SC) than the tree component (TC). We indeed found significant relationships between coffee basal area and forests attributes but we found little evidence to support our size-dependent hypothesis, because coffee was significantly related with two out of six attributes for both tree component (tree density and percentage of pioneer species) and sapling component (density and absolute species richness). Our results contrast with many studies that found null or even positive effects of coffee plants on native tree communities [20, 27, 53], probably because these studies either (1) were conducted in agroforestry systems, that can harbour many native tree species, but most of them are rare species and originated from planting [27], resulting in low species similarity between agroforests and old-growth forests [53]; or (2) compared community attributes between forests with coffee and surrounding native forests, without evaluating the direct relationship between coffee and the native community. The negative relationship between coffee basal area and tree and sapling density could be related to the competition for space and resources. Although this is a common pattern find in the natural forests (i.e. if a given species is abundant in a plot, that the rest of the species are less abundant), our results highlight that coffee competition is by far stronger than its ability to facilitate other species. Coffee reaches reproductive stage (4–5 years) at a size when most native tree species are still investing in growth [56]. Increases in coffee dominance (an understory species with dense crown) may increases light competition in the understory where saplings are growing, reducing the establishment of native species in the sapling component and consequently, density of native trees. We also found a negative relationship between coffee basal area and absolute species richness, although we did not find the same relationship for rarefied species richness (a density-independent metric). It is well known that absolute species richness is directly related to the density of individuals, which indicates that coffee basal area and saplings species diversity should not be directly related. However, the number of native saplings in many plots is more likely closer to the lower part of the species rarefaction curve (rather than to the asymptote) and may be not representative of plots actual species richness). Our results suggest that coffee might have at least an indirect effect on saplings species richness, as a consequence of the decrease in native sapling density.

We hypothesized that coffee, an understory species with rather dense crown, will reduce the percentage of pioneer individuals that have high light requirements for successful regeneration. Contrary to our expectations, coffee basal area was positively related with the percentage of pioneer individuals in the tree component. Similar results were found for the effect of lianas on tropical forest species, where liana density and the density and species richness of pioneer trees were positively correlated [62, 63]. We suggest four likely, but not mutually exclusive, explanations for the positive relationship between coffee basal area and pioneer tree density. First, coffee may share life-history traits with other shade-tolerant species with high dominance (e.g. *Euterpe edulis*, *Pseudopiptadenia contorta* and *Siparuna guianensis*, that mostly occurs where coffee basal area is low, see Fig 2), and are therefore likely to compete strongly than with pioneer species. This negative interaction between coffee and other shade tolerant species could be driving the observed pattern. Second, pioneer species are capable of rapid growth [32], quickly surpassing coffee plants, reaching higher light conditions, and hence, increasing their abundance compared to the slow-growing shade-tolerant species. Third, the positive relationship between coffee and pioneer trees could be driven by the pioneer species instead of by coffee. In the past, the shaded coffee plantations were managed to be shaded by pioneer species such as *Piptadenia gonoacantha* (a nitrogen-fixing species with high representativeness in the community, see Fig 2), that provide good environmental conditions for coffee to grow. Nevertheless, other pioneer species such as *Nectandra nitidula* and *Xylopia sericea*, though not used to shade coffee plantations, have high density in the tree component where coffee is dominant (Fig 2). Finally, the intensive past management and actual landscape configuration (small size of the forest patches and the surrounding urban matrix) might play an important role in the vegetation structure and species composition. Such types of forest degradation may reduce native density and species richness itself, and increase the high overall proportion of pioneer species in the communities by limiting the arrival of seeds from non-pioneer species, and hence, reducing the competition for the same resources used by coffee population.

Increases in the abundance of pioneer trees may have multiple effects on forests structure and species composition. For instance, pioneer species have inherent short lifespan and can lead to high biomass mortality and lower standing biomass stocks [64, 65], reducing forests carbon stocks and residence time. Moreover, canopy gaps are more frequent in forests dominated by pioneer species, leading to changes in species composition that contrasts sharply with that of mature forests [66].

We acknowledge that the observed relationships do not necessarily mean causation, and that the small number of replicates and the experimental design does not allow for strong conclusions about the invasive potential of *Coffea arabica*. However, our study provides important insights for future research in this topic. Given the fact that coffee hardly occurs in secondary forests that were unshaded coffee plantations in the past suggests that coffee may not be considered an strong invasive species, since it cannot persist in areas in which it is not initially planted in its optimal conditions (i.e. shaded environmental). Our findings that coffee is still present in high abundance (accounting for almost half of sampled individuals) in secondary forests from shaded plantations areas after more than 70 years, highlight coffee legacy and persistence in those forests. We also found lower density and diversity of saplings and higher number of pioneer trees where coffee is dominant, suggesting that coffee is a strong competitor in secondary forests, potentially overyielding native species and taking advantage of degraded forests. As a consequence, some important ecological process of secondary forests can be altered, such as species composition, light requirements for regeneration, forest dynamics and aboveground biomass stocks and carbon residence. Moreover, they suggest the need for management of coffee in abandoned shaded coffee plantations because coffee may be negatively related to forest regeneration. Studies on a large-scale will be important to understand the role of this species in such forests and to manage these areas for conservation and sustainable use of agroforestry systems.

## Supporting information

S1 Table Density, basal area (cm^2^), frequency and successional group (P = pioneer; NP = Non-pioneer; Ex = exotic; and NA = Not available) of all species sampled in the tree and sapling component of secondary forest patches (BGJF-1 = shaded area 1, MPP = shaded area 2, and BGJF-2 = unshaded area) regrowing after the abandonment of coffee plantations (>70 years). (XLSX)

S2 Table Coffee density, coffee basal area (cm^2^), native species density, native basal area (cm^2^), native species richness, Rarefied species richness (S’), Shannon diversity index (H’) and percentage of pioneer individuals (P) per plot of tree component (TC) and sapling component (SC) in three study areas (BGJF-1 = shaded area 1; MPP = shaded area 2; and BGJF-2 = unshaded area). Coffee density and basal area were calculated for both TC and SC.
(DOCX)

## References

[1] Chazdon RL, Broadbent EN, Rozendaal DM, Bongers F, Zambrano AMA, Aide TM, et al Carbon sequestration potential of second-growth forest regeneration in the Latin American tropics. Science Advances. 2016;2(5):e1501639 doi: 10.1126/sciadv.1501639 27386528 10.1126/sciadv.1501639 PMC4928921

[2] Letcher SG, Chazdon RL. Rapid recovery of biomass, species richness, and species composition in a forest chronosequence in northeastern Costa Rica. Biotropica. 2009;41(5):608–17.

[3] Chazdon RL. Making tropical succession and landscape reforestation successful. Journal of Sustainable Forestry. 2013;32(7):649–58.

[4] Lugo AE, Helmer E. Emerging forests on abandoned land: Puerto Rico’s new forests. Forest Ecology and Management. 2004;190(2):145–61.

[5] Chazdon RL. Beyond deforestation: restoring forests and ecosystem services on degraded lands. science. 2008;320(5882):1458–60. doi: 10.1126/science.1155365 18556551 10.1126/science.1155365

[6] Peltzer D, Kurokawa H, Wardle DA. Soil fertility and disturbance interact to drive contrasting responses of co-occurring native and non-native species. Ecology. 2015. 10.1890/15-0298.1 27145625

[7] Mello TJ, de Oliveira AA. Making a Bad Situation Worse: An Invasive Species Altering the Balance of Interactions between Local Species. PloS one. 2016;11(3):e0152070 doi: 10.1371/journal.pone.0152070 27010846 10.1371/journal.pone.0152070 PMC4807039

[8] Pyšek P, Jarošík V, Hulme PE, Pergl J, Hejda M, Schaffner U, et al A global assessment of invasive plant impacts on resident species, communities and ecosystems: the interaction of impact measures, invading species' traits and environment. Global Change Biology. 2012;18(5):1725–37.

[9] Simberloff D, Martin J-L, Genovesi P, Maris V, Wardle DA, Aronson J, et al Impacts of biological invasions: what's what and the way forward. Trends in ecology & evolution. 2013;28(1):58–66. 22889499 10.1016/j.tree.2012.07.013

[10] Warren II RJ. Ghosts of cultivation past-Native American dispersal legacy persists in tree distribution. PloS one. 2016;11(3):e0150707 doi: 10.1371/journal.pone.0150707 26982877 10.1371/journal.pone.0150707 PMC4794212

[11] Hulme PE. Beyond control: wider implications for the management of biological invasions. Journal of Applied Ecology. 2006;43(5):835–47.

[12] Baruch Z, Nozawa S. Abandoned coffee plantations: Biodiversity conservation or path for non-native species? case study in a neotropical montane forest. Interciencia. 2014;39(8):554.

[13] Chai SL, Tanner E. 150‐year legacy of land use on tree species composition in old‐secondary forests of Jamaica. Journal of Ecology. 2011;99(1):113–21.

[14] Wakjira FS. Biodiversity and ecology of Afromontane rainforests with wild Coffea arabica L populations in Ethiopia: Cuvillier Verlag; 2006.

[15] Tadesse G, Zavaleta E, Shennan C. Coffee landscapes as refugia for native woody biodiversity as forest loss continues in southwest Ethiopia. Biological Conservation. 2014;169:384–91.

[16] Schroth G, Laderach P, Dempewolf J, Philpott S, Haggar J, Eakin H, et al Towards a climate change adaptation strategy for coffee communities and ecosystems in the Sierra Madre de Chiapas, Mexico. Mitigation and Adaptation Strategies for Global Change. 2009;14(7):605–25.

[17] Hundera K, Aerts R, Fontaine A, Van Mechelen M, Gijbels P, Honnay O, et al Effects of coffee management intensity on composition, structure, and regeneration status of Ethiopian moist evergreen afromontane forests. Environmental management. 2013;51(3):801–9. doi: 10.1007/s00267-012-9976-5 23180249 10.1007/s00267-012-9976-5

[18] Malavolta E. Historia do café no Brasil: Agronomia agricultura e Comercialização: Editora Agronômica Ceres Ltda.; 2000.

[19] Noponen MR, Healey JR, Soto G, Haggar JP. Sink or source—the potential of coffee agroforestry systems to sequester atmospheric CO 2 into soil organic carbon. Agriculture, ecosystems & environment. 2013;175:60–8.

[20] López-Gómez AM, Williams-Linera G, Manson RH. Tree species diversity and vegetation structure in shade coffee farms in Veracruz, Mexico. Agriculture, ecosystems & environment. 2008;124(3):160–72.

[21] Moguel P, Toledo VM. Biodiversity conservation in traditional Coffee Systems of Mexico. Conservation biology: the journal of the Society for Conservation Biology. 1999;13(1):11–21.

[22] Caudill SA, Rice RA. Do Bird Friendly® Coffee Criteria Benefit Mammals? Assessment of Mammal Diversity in Chiapas, Mexico. PloS one. 2016;11(11):e0165662 doi: 10.1371/journal.pone.0165662 27880773 10.1371/journal.pone.0165662 PMC5120788

[23] Richardson DM, Rejmánek M. Trees and shrubs as invasive alien species—a global review. Diversity and Distributions. 2011;17(5):788–809. doi: 10.1111/j.1472-4642.2011.00782.x

[24] Zenni RD, Ziller SR. An overview of invasive plants in Brazil. Brazilian Journal of Botany. 2011;34(3):431–46.

[25] Joshi AA, Mudappa D, Raman TRS. Brewing trouble: coffee invasion in relation to edges and forest structure in tropical rainforest fragments of the Western Ghats, India. Biological Invasions. 2009;11(10):2387–400. doi: 10.1007/s10530-009-9423-6

[26] Perfecto I, Rice RA, Greenberg R, van der Voort ME. Shade Coffee: A Disappearing Refuge for Biodiversity. BioScience. 1996;46(8):598–608. doi: 10.2307/1312989

[27] Häger A, Otárola MF, Stuhlmacher MF, Castillo RA, Arias AC. Effects of management and landscape composition on the diversity and structure of tree species assemblages in coffee agroforests. Agriculture, Ecosystems & Environment. 2015;199:43–51.

[28] Goodall KE, Bacon CM, Mendez VE. Shade tree diversity, carbon sequestration, and epiphyte presence in coffee agroecosystems: A decade of smallholder management in San Ramón, Nicaragua. Agriculture, Ecosystems & Environment. 2015;199:200–6.

[29] Sandor ME, Chazdon RL. Remnant trees affect species composition but not structure of tropical second-growth forest. PloS one. 2014;9(1):e83284 doi: 10.1371/journal.pone.0083284 ; PubMed Central PMCID: PMC3890367. 24454700 10.1371/journal.pone.0083284 PMC3890367

[30] Hu G, Feeley KJ, Yu M. Habitat Fragmentation Drives Plant Community Assembly Processes across Life Stages. PloS one. 2016;11(7):e0159572 doi: 10.1371/journal.pone.0159572 27427960 10.1371/journal.pone.0159572 PMC4948860

[31] Catford JA, Daehler CC, Murphy HT, Sheppard AW, Hardesty BD, Westcott DA, et al The intermediate disturbance hypothesis and plant invasions: Implications for species richness and management. Perspectives in Plant Ecology, Evolution and Systematics. 2012;14(3):231–41.

[32] Poorter L, Bongers L, Bongers F. Architecture of 54 moist-forest tree species: traits, trade-offs, and functional groups. Ecology. 2006;87(5):1289–301. 16761607 10.1890/0012-9658(2006)87[1289:aomtst]2.0.co;2

[33] Bourdier T, Cordonnier T, Kunstler G, Piedallu C, Lagarrigues G, Courbaud B. Tree size inequality reduces forest productivity: an analysis combining inventory data for ten European species and a light competition model. PloS one. 2016;11(3):e0151852 doi: 10.1371/journal.pone.0151852 26999820 10.1371/journal.pone.0151852 PMC4801349

[34] Onoda Y, Saluñga JB, Akutsu K, Aiba Si, Yahara T, Anten NP. Trade‐off between light interception efficiency and light use efficiency: implications for species coexistence in one‐sided light competition. Journal of Ecology. 2014;102(1):167–75.

[35] Gommers CM, Visser EJ, St Onge KR, Voesenek LA, Pierik R. Shade tolerance: when growing tall is not an option. Trends in Plant Science. 2013;18(2):65–71. doi: 10.1016/j.tplants.2012.09.008 23084466 10.1016/j.tplants.2012.09.008

[36] Alvares CA, Stape JL, Sentelhas PC, de Moraes G, Leonardo J, Sparovek G. Köppen's climate classification map for Brazil. Meteorologische Zeitschrift. 2013;22(6):711–28.

[37] Dean W. With broadax and firebrand: the destruction of the Brazilian Atlantic Forest: Univ of California Press; 1997.

[38] Santiago DS, Fonseca CR, Carvalho FA. Fitossociologia da regeneração natural de um fragmento urbano de Floresta Estacional Semidecidual (Juiz de Fora, MG) Brazilian Journal of Agricultural Sciences. 2014;9(1):117–23.

[39] Oliveira-Neto NE, Nascimento DR, Carvalho FA. Biodiversity inventory of trees in a neotropical secondary forest after abandonment of shaded coffee plantation. iForest-Biogeosciences and Forestry. 2017;10(1):303.

[40] Swaine M, Whitmore T. On the definition of ecological species groups in tropical rain forests. Vegetatio. 1988;75(1–2):81–6.

[41] Oliveira-Filho AT, Scolforo JRS. Inventário florestal de Minas Gerais: espécies arbóreas da flora nativa. Lavras: Editora UFLA; 2008. 619p p.

[42] Carreño‐Rocabado G, Peña‐Claros M, Bongers F, Alarcón A, Licona JC, Poorter L. Effects of disturbance intensity on species and functional diversity in a tropical forest. Journal of Ecology. 2012;100(6):1453–63.

[43] Lohbeck M, Lebrija-Trejos E, Martinez-Ramos M, Meave JA, Poorter L, Bongers F. Functional trait strategies of trees in dry and wet tropical forests are similar but differ in their consequences for succession. PloS one. 2015;10(4):e0123741 doi: 10.1371/journal.pone.0123741 ; PubMed Central PMCID: PMC4412708. 25919023 10.1371/journal.pone.0123741 PMC4412708

[44] Kissling WD, Carl G. Spatial autocorrelation and the selection of simultaneous autoregressive models. Global Ecology and Biogeography. 2008;17(1):59–71.

[45] Latta G, Temesgen H, Adams D, Barrett T. Analysis of potential impacts of climate change on forests of the United States Pacific Northwest. Forest Ecology and Management. 2010;259(4):720–9.

[46] F Dormann C, M McPherson J, B Araújo M, Bivand R, Bolliger J, Carl G, et al Methods to account for spatial autocorrelation in the analysis of species distributional data: a review. Ecography. 2007;30(5):609–28.

[47] Gotelli NJ, Colwell RK. Quantifying biodiversity: procedures and pitfalls in the measurement and comparison of species richness. Ecology letters. 2001;4(4):379–91.

[48] Bates D, Maechler M, Bolker B. lme4: Linear mixed-effects models using S4 classes. R package version 0.999375–42; 2011. Reference Source. 2012.

[49] Oksanen J, Blanchet FG, Kindt R, Legendre P, Minchin PR, O’Hara R, et al Package ‘vegan’. Community ecology package, version. 2013;2(9).

[50] Bivand R, Bernat A, Carvalho M, Chun Y, Dormann C, Dray S, et al The spdep package. Comprehensive R Archive Network, Version. 2005:05–83.

[51] Wickham H, Chang W. ggplot2: an implementation of the grammar of graphics, version 2.1. 0. See https://cranr-projectorg/web/packages/ggplot2/index html. 2016.

[52] Barros KART, Raymundo D, Fonseca SN, Ribeiro JHC, Fonseca CR, Almeida VC, et al Estrutura e diversidade da regeneração florestal na nascente do Córrego São Pedro, Juiz de Fora, MG Revista Agrogeoambiental 2015.

[53] Marcano-Vega H, Aide TM, Bàez D. Forest regeneration in abandoned coffee plantations and pastures in the Cordillera Central of Puerto Rico. Plant Ecology. 2002;161:75–87.

[54] Paiva RVEe, Ribeiro JHC, Carvalho FA. Estrutura, Diversidade e heterogeneidade do estrato regenerante em um fragmento florestal urbano após 10 anos de sucessão florestal. Floresta. 2015;45(3):535–44. doi: 10.5380/rf.v45i3.34533

[55] DaMatta FM. Ecophysiological constraints on the production of shaded and unshaded coffee: a review. Field Crops Research. 2004;86(2):99–114.

[56] DaMatta FM. Exploring drought tolerance in coffee: a physiological approach with some insights for plant breeding. Brazilian Journal of Plant Physiology. 2004;16(1):1–6.

[57] DaMatta FM, Ramalho JDC. Impacts of drought and temperature stress on coffee physiology and production: a review. Brazilian Journal of Plant Physiology. 2006;18(1):55–81.

[58] Tully KL, Lawrence D. Canopy and leaf composition drive patterns of nutrient release from pruning residues in a coffee agroforest. Ecological Applications. 2012;22(4):1330–44. 22827139 10.1890/10-2342.1

[59] DaMatta FM, Ronchi CP, Maestri M, Barros RS. Ecophysiology of coffee growth and production. Brazilian journal of plant physiology. 2007;19(4):485–510.

[60] Schnitzer SA, Klironomos JN, HilleRisLambers J, Kinkel LL, Reich PB, Xiao K, et al Soil microbes drive the classic plant diversity–productivity pattern. Ecology. 2011;92(2):296–303. 21618909 10.1890/10-0773.1

[61] De Souza HN, de Goede RG, Brussaard L, Cardoso IM, Duarte EM, Fernandes RB, et al Protective shade, tree diversity and soil properties in coffee agroforestry systems in the Atlantic Rainforest biome. Agriculture, Ecosystems & Environment. 2012;146(1):179–96.

[62] Schnitzer SA, Carson WP. Treefall gaps and the maintenance of species diversity in a tropical forest. Ecology. 2001;82(4):913–9.

[63] Schnitzer SA, Dalling JW, Carson WP. The impact of lianas on tree regeneration in tropical forest canopy gaps: evidence for an alternative pathway of gap‐phase regeneration. Journal of Ecology. 2000;88(4):655–66.

[64] Poorter L, Sande M, Thompson J, Arets E, Alarcón A, Álvarez‐Sánchez J, et al Diversity enhances carbon storage in tropical forests. Global Ecology and Biogeography. 2015;24(11):1314–28.

[65] Sande MT, Poorter L, Kooistra L, Balvanera P, Thonicke K, Thompson J, et al Biodiversity in species, traits, and structure determines carbon stocks and uptake in tropical forests. Biotropica. 2017.

[66] Poorter L, van der Sande MT, Arets EJ, Ascarrunz N, Enquist B, Finegan B, et al Biodiversity and climate determine the functioning of Neotropical forests. Global Ecology and Biogeography. 2017;26(12):1423–34.

