# Supplementary material for: Persistence of Coffea arabica and its relationship with the structure, species diversity and composition of a secondary forest in Brazil
Source: PLoS One. 2018 Mar 14;13(3):e0194032. doi: 10.1371/journal.pone.0194032 (PMC5851612; doi:10.1371/journal.pone.0194032)
Supplement: S2 Table — Coffee density and basal area were calculated for both TC and SC. (DOCX) [file pone.0194032.s002.docx]

S2 Table. Coffee density, coffee basal area (cm²), native species density, native basal area (cm²), native species richness, Rarefied species richness (S’), Shannon diversity index (H’) and percentage of pioneer individuals (P) per plot of tree component (TC) and sapling component (SC) in three study areas (shaded area 1 - BGJF-1, shaded area - 2 MPP, and unshaded area - BGJF-2). Coffee density and basal area were calculated for both TC and SC.

| **Area** | **Plot** | **Coffee density** | **Coffee basal area** | **Density** | | **Basal area** | | **Species richness** | | **S'** | | **H'** | | **P** | |
| --- | --- | --- | --- | --- | --- | --- | --- | --- | --- | --- | --- | --- | --- | --- | --- |
|  |  |  |  | TC | SC | TC | SC | TC | SC | TC | SC | TC | SC | TC | SC |
| MPP | 1 | 16 | 45.6 | 51 | 52 | 25276.6 | 129.5 | 23 | 9 | 7.03 | 3.21 | 2.77 | 1.11 | 66.10 | 73.80 |
| MPP | 2 | 656 | 798.5 | 29 | 46 | 14521.3 | 129.4 | 12 | 12 | 5.34 | 5.13 | 1.93 | 1.95 | 67.70 | 39.80 |
| MPP | 3 | 272 | 693.9 | 41 | 25 | 10701.5 | 48.3 | 20 | 11 | 7.32 | 6.04 | 2.76 | 2.12 | 49.10 | 35.70 |
| MPP | 4 | 448 | 1196.5 | 48 | 29 | 11086.8 | 109.7 | 20 | 8 | 6.01 | 4.82 | 2.38 | 1.74 | 56.90 | 25.00 |
| MPP | 5 | 528 | 1263.2 | 30 | 20 | 4240.5 | 73.9 | 14 | 11 | 5.81 | 6.41 | 2.13 | 2.16 | 51.40 | 14.30 |
| MPP | 6 | 256 | 634.5 | 67 | 15 | 39277.8 | 83.8 | 21 | 5 | 6.68 | 3.91 | 2.65 | 1.21 | 64.30 | 8.60 |
| MPP | 7 | 176 | 339.0 | 38 | 18 | 12902.7 | 89.8 | 12 | 10 | 6.43 | 6.31 | 2.31 | 2.09 | 50.00 | 10.50 |
| MPP | 8 | 384 | 1115.1 | 26 | 18 | 21052.3 | 58.1 | 11 | 10 | 5.67 | 6.03 | 2.03 | 1.98 | 64.30 | 23.30 |
| MPP | 9 | - | - | 62 | 45 | 13316.4 | 77.3 | 32 | 21 | 7.44 | 6.18 | 3.08 | 2.45 | 57.10 | 41.30 |
| MPP | 10 | 16 | 28.8 | 56 | 59 | 19876.7 | 78.5 | 19 | 10 | 6.12 | 3.48 | 2.41 | 1.26 | 68.40 | 16.70 |
| BGJF-1 | 1 | 64 | 208.0 | 73 | 26 | 19340.6 | 93.2 | 24 | 16 | 5.92 | 7.52 | 2.44 | 2.63 | 47.30 | 20.00 |
| BGJF-1 | 2 | 448 | 1711.5 | 86 | 6 | 17432.0 | 25.6 | 23 | 5 | 5.10 | - | 2.14 | 1.56 | 34.80 | 2.90 |
| BGJF-1 | 3 | 528 | 2046.1 | 80 | 11 | 14283.9 | 71.0 | 21 | 10 | 4.81 | 8.35 | 2.00 | 2.27 | 39.50 | 4.50 |
| BGJF-1 | 4 | 304 | 546.9 | 76 | 14 | 15901.8 | 26.0 | 16 | 12 | 4.59 | 8.21 | 1.84 | 2.44 | 34.60 | 9.10 |
| BGJF-1 | 5 | 721 | 1669.4 | 42 | 17 | 15374.5 | 78.8 | 17 | 11 | 6.68 | 6.67 | 2.51 | 2.17 | 55.80 | - |
| BGJF-1 | 6 | - | - | 78 | 9 | 18289.3 | 16.4 | 12 | 3 | 3.10 | - | 1.12 | 0.85 | 20.30 | 11.10 |
| BGJF-1 | 7 | 96 | 115.9 | 90 | 14 | 13769.7 | 118.1 | 15 | 6 | 3.87 | 4.96 | 1.51 | 1.57 | 23.90 | 20.00 |
| BGJF-1 | 8 | 48 | 130.9 | 69 | 18 | 13384.3 | 65.6 | 21 | 14 | 4.94 | 8.06 | 2.03 | 2.58 | 22.90 | 36.40 |
| BGJF-1 | 9 | 260 | 1070.6 | 54 | 15 | 10480.5 | 64.1 | 23 | 11 | 6.19 | 7.47 | 2.51 | 2.30 | 31.10 | 12.90 |
| BGJF-1 | 10 | 628 | 1469.5 | 55 | 3 | 26902.3 | 31.0 | 15 | 2 | 4.82 | - | 1.88 | 0.64 | 24.30 | 4.80 |
| BGJF-1 | 11 | 224 | 749.5 | 77 | 8 | 20268.1 | 57.1 | 23 | 7 | 5.13 | - | 2.13 | 1.91 | 32.90 | 13.60 |
| BGJF-1 | 12 | 321 | 1387.6 | 60 | 9 | 18039.9 | 70.9 | 27 | 6 | 6.27 | 6.00 | 2.61 | 1.68 | 34.40 | - |
| BGJF-1 | 13 | 515 | 1960.4 | 55 | 4 | 21677.7 | 60.9 | 16 | 4 | 4.66 | - | 1.83 | 1.39 | 27.10 | - |
| BGJF-1 | 14 | 145 | 584.8 | 63 | 32 | 14751.9 | 215.4 | 19 | 14 | 5.10 | 6.45 | 2.05 | 2.34 | 35.90 | 4.90 |
| BGJF-1 | 15 | 305 | 1067.2 | 61 | 14 | 18951.3 | 92.6 | 24 | 8 | 5.51 | 5.75 | 2.29 | 1.77 | 38.10 | 26.50 |
| BGJF-1 | 16 | 403 | 975.1 | 49 | 22 | 17275.4 | 145.2 | 19 | 13 | 6.14 | 7.03 | 2.42 | 2.39 | 47.20 | 6.40 |
| BGJF-1 | 17 | 387 | 2248.3 | 72 | 5 | 18105.5 | 17.4 | 24 | 5 | 5.42 | - | 2.26 | 1.61 | 30.70 | 3.40 |
| BGJF-1 | 18 | 260 | 1171.2 | 70 | 8 | 10531.0 | 42.4 | 24 | 6 | 5.91 | - | 2.44 | 1.67 | 33.80 | 8.30 |
| BGJF-1 | 19 | 628 | 2222.0 | 49 | 9 | 12326.9 | 46.3 | 20 | 3 | 6.40 | 3.00 | 2.51 | 0.94 | 32.70 | - |
| BGJF-1 | 20 | 64 | 185.2 | 77 | 8 | 16065.6 | 42.6 | 20 | 6 | 4.53 | - | 1.85 | 1.67 | 28.00 | 7.70 |
| BGJF-1 | 21 | 113 | 251.7 | 79 | 9 | 17597.5 | 40.5 | 20 | 6 | 4.62 | 6.00 | 1.89 | 1.68 | 25.90 | 6.30 |
| BGJF-1 | 22 | 336 | 2201.4 | 73 | 29 | 19219.0 | 191.3 | 20 | 10 | 5.38 | 5.60 | 2.18 | 2.00 | 45.20 | 8.00 |
| BGJF-1 | 23 | 498 | 953.5 | 58 | 7 | 20763.3 | 73.0 | 22 | 5 | 5.93 | - | 2.40 | 1.55 | 51.70 | 7.90 |
| BGJF-1 | 24 | 144 | 304.6 | 63 | 29 | 16677.1 | 88.4 | 24 | 11 | 6.98 | 5.62 | 2.80 | 2.03 | 37.70 | 23.70 |
| BGJF-1 | 25 | 304 | 687.9 | 76 | 7 | 21756.5 | 22.7 | 19 | 7 | 5.75 | - | 2.30 | 1.95 | 46.30 | 22.20 |
| BGJF-2 | 1 | - | - | 116 | 49 | 8682.5 | 112.4 | 23 | 14 | 5.82 | 4.45 | 2.39 | 1.70 | 64.50 | 86.00 |
| BGJF-2 | 2 | - | - | 95 | 35 | 7500.6 | 126.0 | 14 | 16 | 5.76 | 6.66 | 2.22 | 2.46 | 67.00 | 45.90 |
| BGJF-2 | 3 | - | - | 89 | 31 | 8551.4 | 99.2 | 16 | 14 | 6.17 | 6.79 | 2.39 | 2.43 | 55.40 | 51.50 |
| BGJF-2 | 4 | - | - | 73 | 55 | 5447.7 | 185.4 | 25 | 16 | 6.54 | 5.65 | 2.67 | 2.20 | 43.80 | 77.60 |
| BGJF-2 | 5 | - | - | 91 | 30 | 6652.0 | 110.9 | 17 | 13 | 5.80 | 6.05 | 2.27 | 2.20 | 69.80 | 57.60 |
| BGJF-2 | 6 | - | - | 99 | 35 | 6468.8 | 55.7 | 19 | 12 | 5.77 | 6.38 | 2.30 | 2.29 | 69.70 | 31.70 |
| BGJF-2 | 7 | - | - | 80 | 53 | 6860.1 | 151.7 | 15 | 17 | 5.68 | 6.74 | 2.20 | 2.56 | 67.10 | 45.60 |
| BGJF-2 | 8 | - | - | 85 | 42 | 6577.8 | 85.2 | 14 | 19 | 5.21 | 6.91 | 2.03 | 2.62 | 67.40 | 38.20 |
| BGJF-2 | 9 | - | - | 82 | 30 | 7616.7 | 81.3 | 15 | 13 | 4.96 | 5.78 | 1.95 | 2.10 | 68.10 | 62.50 |
| BGJF-2 | 10 | 32 | 9.3 | 71 | 44 | 9313.1 | 125.9 | 24 | 17 | 6.66 | 6.50 | 2.69 | 2.47 | 65.80 | 58.70 |
| BGJF-2 | 11 | - | - | 64 | 40 | 5560.8 | 135.9 | 15 | 15 | 6.06 | 6.21 | 2.33 | 2.32 | 64.60 | 62.50 |
| BGJF-2 | 12 | - | - | 59 | 44 | 7648.5 | 82.8 | 12 | 18 | 4.76 | 7.10 | 1.83 | 2.67 | 61.00 | 51.00 |
| BGJF-2 | 13 | - | - | 68 | 30 | 4478.1 | 97.3 | 16 | 15 | 5.40 | 7.21 | 2.12 | 2.56 | 62.30 | 51.40 |
| BGJF-2 | 14 | - | - | 69 | 47 | 4904.3 | 109.6 | 12 | 16 | 4.53 | 6.92 | 1.73 | 2.57 | 69.10 | 56.90 |
| BGJF-2 | 15 | - | - | 112 | 35 | 8537.1 | 102.5 | 18 | 10 | 5.30 | 4.60 | 2.14 | 1.71 | 75.20 | 77.10 |
| BGJF-2 | 16 | - | - | 66 | 32 | 5364.3 | 59.5 | 17 | 14 | 6.09 | 6.68 | 2.38 | 2.40 | 64.80 | 73.50 |
| BGJF-2 | 17 | - | - | 62 | 54 | 5858.0 | 136.9 | 12 | 17 | 4.60 | 6.44 | 1.80 | 2.47 | 86.20 | 65.50 |
| BGJF-2 | 18 | - | - | 56 | 29 | 12518.2 | 85.3 | 8 | 13 | 3.11 | 6.25 | 1.06 | 2.25 | 86.20 | 65.60 |
| BGJF-2 | 19 | - | - | 46 | 56 | 4572.6 | 92.7 | 13 | 14 | 4.80 | 5.60 | 1.82 | 2.15 | 77.80 | 75.90 |
| BGJF-2 | 20 | 288 | 133.8 | 38 | 43 | 9520.5 | 133.3 | 6 | 3 | 2.89 | 2.18 | 1.00 | 0.71 | 74.40 | 70.50 |
| BGJF-2 | 21 | - | - | 32 | 31 | 17208.3 | 81.2 | 4 | 6 | 2.99 | 3.43 | 1.02 | 1.15 | 82.40 | 93.50 |
| BGJF-2 | 22 | - | - | 23 | 52 | 6362.6 | 40.8 | 6 | 8 | 4.19 | 3.56 | 1.46 | 1.25 | 88.00 | 94.20 |
| BGJF-2 | 23 | - | - | 49 | 5 | 6384.2 | 17.1 | 15 | 2 | 5.98 | 0.00 | 2.28 | 0.50 | 59.70 | 20.00 |
| BGJF-2 | 24 | 16 | 10.2 | 82 | 20 | 7344.3 | 52.5 | 19 | 13 | 5.77 | 7.44 | 2.32 | 2.46 | 67.00 | 65.20 |
| BGJF-2 | 25 | - | - | 69 | 20 | 4084.9 | 120.8 | 14 | 11 | 5.06 | 6.70 | 1.97 | 2.23 | 57.60 | 50.00 |

Online Resource available from: Persistence of *Coffea arabica* and its relationship with the structure, species diversity and composition of a secondary forest in Brazil. D Raymundo, JA Prado-Junior, NE Oliveira-Neto, LD Santana, VS Vale, TKB Jacobson, PEAM Oliveira, FA Carvalho.
